# Commensal bacteria contribute to the growth of multidrug-resistant *Avibacterium paragallinarum* in chickens

**DOI:** 10.3389/fmicb.2022.1010584

**Published:** 2022-11-04

**Authors:** Jiajia Zhu, Yunsheng Chen, Yifan Wu, Yongqiang Wang, Kui Zhu

**Affiliations:** ^1^National Center for Veterinary Drug Safety Evaluation, College of Veterinary Medicine, China Agricultural University, Beijing, China; ^2^Guangdong Laboratory for Lingnan Modern Agriculture, Guangzhou, China; ^3^Key Laboratory of Animal Epidemiology of the Ministry of Agriculture and Rural Affairs, College of Veterinary Medicine, China Agricultural University, Beijing, China

**Keywords:** *Avibacterium paragallinarum*, commensal bacteria, satellitism, antimicrobial resistance, symbiosis

## Abstract

*Avibacterium paragallinarum*-associated infectious coryza (IC) is an important threat in commercial poultry. Previous studies about the characteristics of *A. paragallinarum* are succeeded in revealing the course of IC disease, but whether and how resident microbes contribute to the infection remains unclear. To understand the role of commensal bacteria, we isolated 467 commensal bacteria, including 38 *A. paragallinarum*, from the respiratory tract of IC chicken. The predominant commensal isolates were Gram-positive bacteria belonging to *Staphylococcus* spp. [33.19%, 95% confidence interval (CI): 28.93–37.66%], *Enterococcus* spp. (16.49%, 95% CI: 13.23–20.17%), and *Bacillus* spp. (16.27%, 95% CI: 13.04–19.94%). These isolates were closely correlated with the survival of *A. paragallinarum*. We examined and found that commensal bacteria aggravate *A. paragallinarum*-associated infections because certain commensal species (28.57%, 95% CI: 15.72–44.58%) induced hemolysis and promoted the growth of *A. paragallinarum in vitro*. Notably, *A. paragallinarum* showed high resistance to routine antibiotics such as erythromycin (84.21%, 95% CI: 68.75–93.98%), tetracycline (73.68%, 95% CI: 56.90–86.60%) and carried diverse mobile resistance gene clusters. Overall, we found commensal bacteria especially Gram-positive bacteria facilitate the survival of multidrug-resistant *A. paragallinarum* to exacerbate infections, suggesting that novel strategies may diminish *A. paragallinarum*-associated infections by modulating the population dynamics of commensal bacteria.

## Graphical Abstract

The scheme of interactions in this study. 
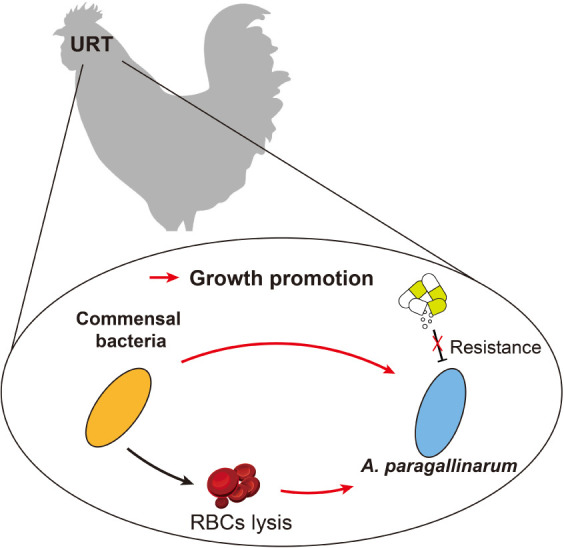

## HIGHLIGHTS

- Commensal bacteria isolated from respiratory tracts in IC chicken show correlative to the presence of *A. paragallinarum* and facilitate the survival.

- The symbiotic relationship between commensals and *A. paragallinarum* commonly present in respiratory tracts of IC chicken.

- Mobile resistance gene clusters are distributed globally in *A. paragallinarum* isolates, against frequently-used antimicrobial agents in commercial poultry.

## Introduction

Infectious coryza (IC) is an important threat to global animal health and causes huge economic losses, resulting in seriously decreased egg production and increased mortality in laying hens (Alvarez et al., 2020; Nouri et al., 2021). The distinguishing clinical symptoms are anorexia, facial swelling, nasal discharge, lacrimation, and even mortality (Xu et al., 2019). IC is an acute upper respiratory tract (URT) inflammation in chickens caused by a specific opportunistic pathogen, *Avibacterium paragallinarum*, historically named *Haemophilus paragallinarum* (Guo et al., 2022). Such bacterial pathogens are nutrient-dependent symbiotic bacteria, especially requiring sufficient accessory growth factors [X, hemin and V, nicotinamide adenine dinucleotide (NAD)] to proliferate *in vivo* (Mouahid et al., 1992). In homeostasis, commensal bacteria exclude exogenous microorganisms and directly inhibit the growth of pathogens (Clark, 2020) resulting in the limited establishment of opportunistic pathogens such as *A. paragallinarum* in respiratory tract (Long et al., 2012). When host immunity is compromised, *A. paragallinarum* initiates colonization and invasion, which is facilitated by commensals like *Gallibacterium anatis* (Paudel et al., 2017), *Ornithobacterium rhinotracheale* (Morales-Erasto et al., 2016), and *Staphylococcus chromogenes* (Wu et al., 2021). Resident species constitute a new niche to allow *A. paragallinarum* colonization and virulence by providing public goods and disrupting host barriers (Hoare et al., 2021; Wu et al., 2021). Additionally, protection mediated by indigenous microbial communities including extensive horizontal gene transfer (McInnes et al., 2020) and antibiotic inactivation (Gjonbalaj et al., 2020) increases the antimicrobial tolerance and resistance of pathogens. The benefit of commensal protection and facilitation is not confined to early growth, rather it is continuous during the infection. As a result, antimicrobials might not perform well in IC disease because of the combined effect within bacterial communities.

As is found that symbiotic interaction is critical for the survival of *A. paragallinarum*, multiple resistance phenotypes emerging in *A. paragallinarum* also contribute to the establishment *in vivo* and limit the efficacy of antimicrobials. To date, vaccination is still the most important implementation to provide effective protection against infection (Van den Biggelaar et al., 2020). However, this intervention is not sufficient, especially for infecting with high virulence and resistance strains (Trujillo-Ruiz et al., 2016). Once the vaccination is no longer effective, antimicrobial is considered as a complementary alternative to alleviate the symptom of the disease. However, irrational use of antibiotics accelerates the development of multidrug resistance and virulence in both pathogens like *A. paragallinarum* and indigenous microbiota (Nhung et al., 2017; Wongsuvan et al., 2018; Juricova et al., 2021). Further, published data about the genetic environment and transferability of virulence genes (VGs) and antimicrobial resistance genes (ARGs) in *A. paragallinarum* are limited. Therefore, characterization of resistant traits of *A. paragallinarum* is urgently needed for the appropriate use of antibiotics.

Although the causative agent of IC disease was defined, further efforts are still required to uncover the correlation between commensals and *A. paragallinarum*. Besides, the resistance genotypes and phenotypes of *A. paragallinarum* are needed to characterize for development of more effective treatment. In this study, we aimed to determine the promotion effect contributed by commensals through find out the association between *A. paragallinarum* and commensal bacteria in URT from IC chickens and subsequently analyzed the characteristics of antimicrobial resistance and virulence in all *A. paragallinarum* isolates to comprehensively understand the traits of resistant *A. paragallinarum*.

## Materials and methods

### Isolation and identification of respiratory tract bacteria

Layer chickens with symptom of IC (*n* = 38) and healthy chickens (*n* = 9) were collected from commercial layer farms in Tianjin, Hebei, and Guangxi in China from 2019 to 2022. All samples were vaccinated against IC and with no antibiotic treatment after infection. The heads of infected and non-infected birds were disinfected with 75% ethyl alcohol before bacterial isolation. Swab samples collected from nasal cavity and infraorbital sinuses were cultured on blood agar [trypticase soya agar (TSA) supplemented with 5% filter-sterilized sheep] and TSA plus agar (supplemented with 20 μg/ml NAD and 5% fetal bovine serum), respectively. TSA plus agar plates were used to isolate the *A. paragallinarum* and incubated aerobically at 5% CO_2_ and 37°C for 24–48 h. The colony morphology consisting with translucent light blue was selected as *A. paragallinarum* for further study. Commensals were cultured in blood agar plates at 37°C for 24 h and further purified in TSA agar. Afterward, all isolates were identified based on MALDI-TOF-MS identification and 16S rRNA gene sequence analysis. After confirming the taxonomic classification of these isolates, *A. paragallinarum* was freeze-dried with 10% skim milk and commensals were maintained in nutrient broth supplemented with 20% glycerol and stored at −80°C.

### Growth-promoting effects of commensals on *Avibacterium paragallinarum* growth

The growth-promotion effect contributed by commensal was determined through the observation of satellitic growth of *A. paragallinarum*. All commensal bacteria were cultured overnight and then diluted to 10^8^ CFUs/ml. Meanwhile, *A. paragallinarum* was cultured in TBS plus broth for 18 h at 37°C, and then diluted to 10^6^ CFUs/ml. Subsequently, 100 μl of diluted bacteria suspension was spread on the TSA blood agar, and then spotted 2 μl of the commensal bacteria solution on the center of the blood plate incubating in carbon dioxide incubator for 24–48 h. The experiment was repeated twice and the radius of growing circle of *A. paragallinarum* was recorded. The calculation equation of promotion rate = growth radius of *A. paragallinarum* − growth radius of commensal.

According to the previous research (Wu et al., 2021), the relationship between *A. paragallinarum* and commensals was defined as three synergistic indexes by the following formulae: (1) Isolation rate (%) = the number of isolates of a specific genus or species/total number of isolates; (2) Satellitism rate (%) = the number of specific species or genus of bacteria that facilitate the growth of *A. paragallinarum*/total number of commensal isolates that facilitate the growth of *A. paragallinarum*; (3) Colocalization rate (%) = the number of chickens infected with a specific species or genus of bacteria and *A. paragallinarum*/the number of chickens infected with *A. paragallinarum*.

### Hemolysis detection of commensals

To evaluate the hemolytic activity of commensal bacteria, isolates were spot on 5% sheep blood cells (RBCs) TSA agar after diluted to 10^6^ CFUs/ml, and then recorded the transparent hemolysis radius after cultured for 12, 24, 36, 48, 60, and 72 h at 37°C. Meanwhile, hemolysis of supernatant from commensals was also determined according to the method described previously (Deng et al., 2021) with minor modifications. In brief, isolates were cultured in TSB for 24, 48, and 72 h to harvest the bacterial supernatant. RBCs were harvested by centrifugation (1,000 rpm/min, 10 min) and rinsed three times in phosphate buffered saline (PBS) solution. After RBCs was diluted to 8% (vol/vol), 100 μl bacterial supernatant was mixed with an equal volume of 8% RBCs and incubated at 37°C for 1 h, and then centrifuged at 1,000 rpm for 10 min at 4°C. The 4% RBCs suspension in PBS and Triton X-100 (0.1%) with RBCs suspension were used as negative control and positive control, respectively. The hemolytic activity was assessed by measuring the optical absorbance of the supernatant at OD_576_. The calculation equation of hemolysis: Hemolysis rate (%) = (OD_Sample_ − OD_Negative_)/(OD_Positive_ − OD_Negative_) × 100%.

### Antimicrobial susceptibility test

The broth microdilution method, as described by the Clinical and Laboratory Standards Institute guidelines (Clinical and Laboratory Standards Institute [CLSI], 2020), was used to determine the susceptibility of 38 *A. paragallinarum* isolates to 13 antimicrobial agents (the categories of antimicrobial agents were shown in Supplementary Table 2). The medium used in the susceptibility test was supplemented with 10% chicken serum and 0.0025% NAD (CAMHB plus broth) as described previously (Heuvelink et al., 2018). The MIC (minimum inhibitory concentration) ranges and breakpoints for ampicillin, erythromycin, and tetracycline were found *via* referral to the previous research (Blackall, 1988). For other antimicrobial agents (penicillin and meropenem), these criteria were referred to CLSI documents (Clinical and Laboratory Standards Institute [CLSI], 2016). *Escherichia coli* ATCC 25922 served as the quality control strain to validate the results obtained.

### Whole-genome sequencing and analysis of *Avibacterium paragallinarum*

Genomic DNA of *A. paragallinarum* was prepared by using the TIANamp Bacterial DNA Kit (Tiangen Biotech, Beijing, China). DNA was then fragmented to prepare the library and was sequenced using Illumina NovaSeq 6,000 (Illumina, United States) in pair-end model. High-quality reads were *de novo* assembled by using SPAdes v3.12.0 (Bankevich et al., 2012) and annotated by Prokka v1.14.6 (Seemann, 2014). Phylogenetic relationships between the isolates were determined by bacterial core genome using bcgTree (Ankenbrand and Keller, 2016), and SNP analysis was conducted by Harvest v1.1.2 (Treangen et al., 2014). Mobile gene elements (MGEs) were screened including insertion sequences, prophage elements, integrative and conjugative elements (ICEs), and plasmid sequences. ISfinder (Siguier et al., 2006) and ICEberg (Liu et al., 2019) databases were used to identify complete or partial elements *via* BLASTn with detailed parameters (identity ≥70% and coverage ≥50%). Prophages and prophage-like elements were analyzed by Phigaro v2.3.0 (Starikova et al., 2020). The virulence factors and ARGs of *A. paragallinarum* were identified based on VFDB (Chen et al., 2005), ResFinder (Bortolaia et al., 2020), and the Comprehensive Antibiotic Resistance (CARD) (Alcock et al., 2020) database using BLASTn with a cut-off of 60% of coverage and 80% of identity. Finally, the genetic environment of VGs and ARGs gene clusters was further inspected by using CD-search and ORFFinder (Sayers et al., 2022), and collinearity analysis using clinker and clustermap.js (Gilchrist and Chooi, 2021) to compare the similarity of gene clusters with representative gene clusters previously reported.

### Statistical analysis

The data was statistically analyzed by using GraphPad Prism 9. Tests of normal distribution were performed (Shapiro–Wilk test) and data were analyzed using one-way non-parametric (Kruskal–Wallis test) or one-way ANOVA with Tukey’s *post hoc* tests where applicable for continuous variables. LM was performed in the R package (version 4.1.3) to examine the correlation.

## Results

### Composition and diversity of respiratory tract microbiota

**FIGURE 1:**
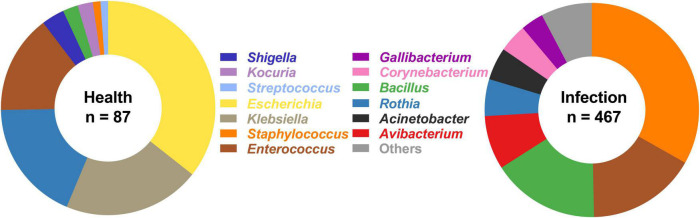
Bacterial composition in upper respiratory tract from healthy chickens and IC chickens. In infection group, the bacterial genera with relative abundance of less than 2% were categorized into “Others” group, including *Aerococcus* spp. (0.21%), *Carnobacterium* spp. (0.21%), *Enterobacter* spp. (0.64%), *Escherichia* spp. (0.21%), *Lysinibacillus* spp. (1.71%), *Paenibacillus* spp. (0.21%), *Streptococcus* spp. (1.07%), *Stenotrophomonas* spp. (0.43%), *Vibrio* spp. (0.21%), and *Kocuria* spp. (1.71%).

A total of 554 culturable bacterial isolates were identified from the respiratory tracts of healthy chickens and IC chickens (Figure 1). The URT bacterial composition in either IC chickens or healthy chickens was generally dominated by bacteria from two different phyla: Firmicutes and Proteobacteria (Supplementary Tables 1, 2). In healthy chickens, Proteobacteria was the predominant phylum, accounting for ∼60% of total isolates. By contrast, the diversity of culturable microbiome was significantly changed when the chicken was infected with *A. paragallinarum*, as Firmicutes were the most prominent phylum. In URT of IC chickens, a total of 467 commensal and pathogenic bacteria were to be isolated, including the causative agents such as *A. paragallinarum*, *G. anatis*, *Pseudomonas aeruginosa*, and *Rothia nasimurium* (Supplementary Table 1). Although *A. paragallinarum* is the primary etiologic agent in IC, most of the isolates cannot survive after being isolated from the host, resulting in a low isolation rate (57.89%, 22/38). In contrast to the healthy group, URT microbiota in infected chickens was mainly composed of Gram-positive bacteria, which was dominated by three most prominent genera: *Staphylococcus* (33.19%), *Enterococcus* (16.49%), and *Bacillus* (16.27%) (Figure 1). Each abundant genera had the predominant species, such as *S. chromogenes*, *E. faecalis*, and *B. subtilis*, with isolation rates of 19.7, 13.06, and 8.14%, respectively (Supplementary Table 1). Interestingly, the bacterial community in URT comprised large amounts of *Bacillus* spp. such as *B. subtilis, B. haynesii, B. amyloliquefaciens*, and *B. velezensis* (Supplementary Table 1), which were rarely reported in previous studies. Some oral and respiratory tract flora were also identified including *Rothia* spp., *Acinetobacter* spp., *Corynebacterium* spp., and *G. anatis*, with isolation rates of 5.57% (26/467), 4.91% (23/467), 4.28% (20/467), and 3.43% (16/467), respectively. The abundance of minor bacterial genera (less than 2% isolation rate) ranging from 1.71 to 0.21% contained respiratory tract pathogens such as *Streptococcus pluranimalium* and *P. aeruginosa*, implying that these isolates might not be the causative agents of IC.

### Commensal bacteria facilitated the survival of *Avibacterium paragallinarum*

**FIGURE 2:**
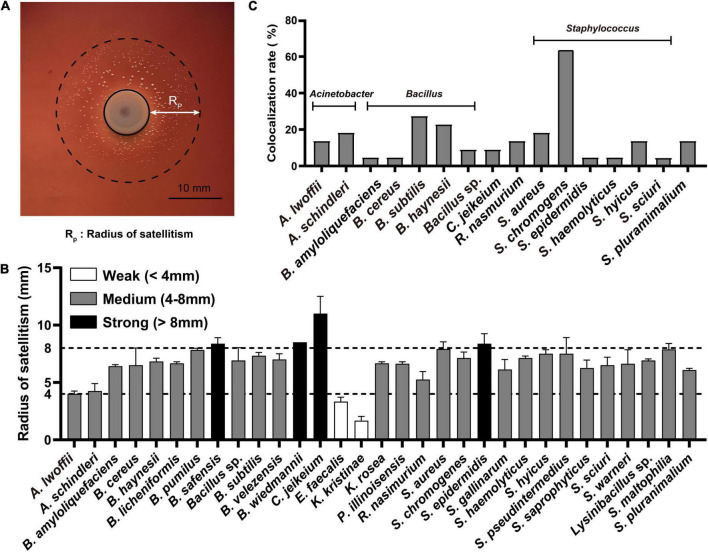
Commensals facilitated the growth of *A. paragallinarum*. **(A)** Representative image of the satellitism that commensal bacterium was surrounded by *A. paragallinarum.* **(B)** The satellitism profiles of commensals from IC chicken. **(C)** The colocalization profiles of commensals with strong growth-promoting effect on *A. paragallinarum*. The full names of genera were replaced by the abbreviation in this figure, and the full name of genera was listed in Supplementary Table 1.

The symbiotic growth of *A. paragallinarum* was associated with a part of commensals. To reveal the potential contribution of commensal to *A. paragallinarum-*associated infections, we determined the growth-promoting ability of commensal bacteria by measuring the radius of satellitic zone (Figure 2A). Based on the radius of satellitism, the strengths of promotion effect were categorized into three degrees: weak (<4 mm), medium (4–8 mm), and strong (>8 mm) (Figure 2B). In URT of IC chickens, the majority of bacterial members (31/42) showed growth-promoting effects on *A. paragallinarum* among which four species exhibited strong promotion, including *B. safensis, B. wiedmanii, C. jeikeium*, and *S. epidermidis*. The ability and strength of growth promotion showed species specificity in commensal bacteria. For instance, *C. jeikeium* was the particular species facilitating the growth of *A. paragallinarum* in genus of *Corynebacterium* spp. *E. faecalis* and *K. kristinae* displayed weak capability of promotion, and *E. faecalis* was the unique species that could interact with *A. paragallinarum* among enterococci. Similarly, in relatively low abundant genera (*Rothia*, *Kocuria*, and *Paenibacillus*), the promotion effect was restricted within certain species. In this case, the ability of growth promotion was not shared by closely related species. Conversely, in two abundant genera (*Bacillus* and *Staphylococcus*), all isolates showed promotion capacity with average satellitic radii ranging from 5 to 9 mm (Figure 2B), suggesting that at least some genera might have common capability that facilitated the survival of *A. paragallinarum*. Importantly, opportunistic pathogens in respiratory tract such as *S. pluranimalium* and *Stenotrophomonas maltophilia* were positive for the result of satellitism, implying that such pathogens may increase the possibility of co-infection in IC chicken.

The interaction between microbes is spatial short-range in communities (Dal Co et al., 2020), thus colocalization is a prerequisite synergistic index that commensals could contribute to the growth of *A. paragallinarum*. A total of 16 species were selected as candidates for further study as they showed strong or medium growth promotion effects and colocalized with *A. paragallinarum* (Figure 2C). It was notable that the genera of *Bacillus* and *Staphylococcus* were the prominent members co-isolating with *A. paragallinarum* and facilitating the survival of *A. paragallinarum in vitro*. In those candidate species, *S. chromogenes* showed extremely colocalized with *A. paragallinarum*. Interestingly, Gram-positive bacteria accounted for nearly 92% of all growth-promotion commensals, suggesting that Gram-positive commensals are primary microorganisms in feeding Gram-negative *A. paragallinarum*.

### Positive correlation among bacterial isolation, colocalization, and satellitism in infectious coryza chickens

**FIGURE 3:**
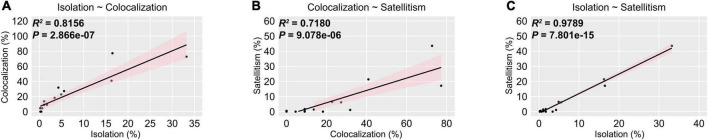
Isolation is positively correlated with co-colonization and satellitism at genus-level. The pink shaded areas represented the 95% confidence intervals. **(A)** Isolation is positively correlated with colocalization; **(B)** Colocalization is positively correlated with satellitism; **(C)** Isolation is positively correlated with satellitism.

Given that interspecific interplays are common in commensal microbiota, it may contribute to flourishment of specialized kinds of commensals in IC chicken, thus we performed correlation analysis using linear regression model to investigate the relationship among bacterial isolation, colocalization, and satellitism. The *p*-value of correlation between isolation and colocalization at genus-level is less than 0.05, with Pearson’s correlation coefficient of 0.8156, indicating that positive correlation between colocalization and isolation irrespective of bacterial taxonomy (Figure 3A). Hence, we hypothesized that isolation and colocalization are closely associated with satellitism in IC chicken. To verify this hypothesis, we calculated the satellite rate in different genus isolates and examined the correlation between satellitism and isolation or colocalization by linear model. The results indicated that satellitism was significant positively correlated with isolation (*p* = 6.334e−08, *R* = 0.847) and colocalization (*p* = 0.001711, *R* = 0.4691) (Figures 3B,C). Of three predominant genera (*Bacillus*, *Enterococcus*, and *Staphylococcus*), the same positive correlations were observed as most of these isolates could support the growth of *A. paragallinarum* (Supplementary Figure 1), but such growth-promoting characteristics were slightly different in *Enterococcus* because the promotion effect was restricted in the specific species *E. faecalis* (Supplementary Table 1). The most noteworthy feature of these correlations was that abundant commensals in URT commonly colocalized with *A. paragallinarum* and could facilitate the survival of *A. paragallinarum* (Supplementary Table 1). In conclusion, the growth-promoting commensals showed positive interaction between *A. paragallinarum*, resulting in richness of promoting commensal bacteria.

### Commensal-mediated hemolysis promoted *Avibacterium paragallinarum* growth

**FIGURE 4:**
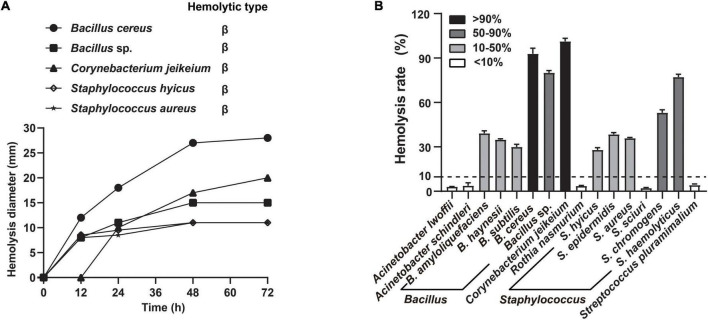
Commensals exhibited extensive hemolytic activity. The selected commensal bacteria were both exhibiting strong or medium growth-promoting effects and colocalizing with *A. paragallinarum*. **(A)** Five representative species were selected to determine the change of hemolysis on blood agar at 12, 24, 48, and 72 h; **(B)** distribution of hemolytic activity among 16 species commensals in supernatant test at 48 h. Hemolysis rate greater than 10% is considered positive hemolysis in this assay.

To obtain nutrients from host, many respiratory tract microbes evolve the ability to disrupt the host barrier, particularly through hemolysis. Notably, the growth factors required by *A. paragallinarum* are available after microbiota-mediated RBCs lysis (Demarest et al., 2019). Thus, we screened the hemolytic activity of candidate commensals under different cultural conditions, to emphasize host-commensal interaction that benefits the *A. paragallinarum* survival. Five of these commensal species exhibited increasing hemolytic activity when cultured in blood agar (Figure 4A). Compared to the solid culture, more commensal species showed hemolysis to various extents in broth medium, such as the complete hemolysis in *B. cereus* and *C. jeikeium*, 50–90% of hemolysis rate in *Bacillus* sp., *S. chromogens*, and *S. haemolyticus* (Figure 4B and Supplementary Figure 2). These tendencies of hemolysis changes are different among colocalized commensals. Certain species (37.5%, 6/16) showed the highest hemolysis rate at 48 h, while 25% (4/16) and 18.75% (3/16) of tested species exhibited the strongest hemolysis at 24 and 72 h, respectively (Supplementary Figure 2). In contrast to static solid cultivation, more species showed hemolysis in liquid shake culture. Delving into more detail, seven species, including *B. amyloliquefaciens*, *B. haynesii*, *B. subtilis*, *R. nasimurium*, *S. epidermidis*, *S. chromogenes*, and *S. haemolyticus*, were positive for hemolysis in liquid cultures rather than in solid cultures, suggesting commensal-mediated hemolysis were prone to potentiate in liquid environment of host cell.

### Antimicrobial resistance in *Avibacterium paragallinarum* compromised antibiotic efficacy

**FIGURE 5:**
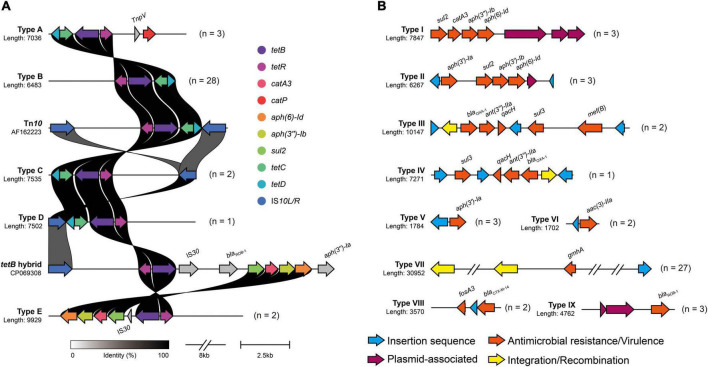
Genetic environment of mobile virulence and antimicrobial resistance gene in *A. paragallinarum*. Different types of antimicrobial resistance gene clusters were shown in above figure, and numbers followed by genetic profile represented the total numbers of similar gene clusters in all *A. paragallinarum*. **(A)** Profiles of *tetB* cluster in *A. paragallinarum*. The major functional proteins in Tn*10* transposon, including TetB (tetracycline efflux protein), TetR (TetR family transcriptional regulator), TetC (transposon Tn*10* TetC protein), TetD (transposon Tn*10* TetD protein), and two insertion sequence IS*10L* and IS*10R*. Tn*10* transposon in *Shigella flexneri* (accession: AF162223) and *tetB* hybrid sequence in *Glaesserella parasuis* (accession: CP069308) served as reference sequences for the comparison of tetracycline resistance gene cluster in *A. paragallinarum.* The gray shading genes indicated that these determinants were dissimilarity among other sequences. **(B)** Diversity of mobile gene cluster in *A. paragallinarum*. Mobile gene clusters excluding *tetB* clusters were categorized into nine sequence types.

In the context of the commensal microbiota, resistant *A. paragallinarum* was promoted to colonize *in vivo*. The resistance characteristics in *A. paragallinarum* isolates further contribute to survival under antibiotic treatment. We found that *A. paragallinarum* isolates showed high MICs values to routine used antibiotics in poultry (Supplementary Table 2). Majority of isolates (73.68%) were resistant to tetracycline, consisting with the presence of tetracycline resistance gene *tetB*. The *tetB* resistance gene was located in Tn*10* transposons and surrounded by mobile elements (Figure 5A). The Tn*10* transposons in *A. paragallinarum* isolates were categorized into five representative sequence types on the basis of genetic environment. We noticed that the novel *tetB* hybrid cluster (Type E) was composed of multiple ARGs such as *cat*, *aph*, and *sul*, counting as Tn*10* variant (Figure 5A). Except for tetracyclines, three kinds of antimicrobials are usually used in the treatment of IC, including macrolides, fluoroquinolones, and β-lactams. In terms of macrolides, 84.21% (32/38) of isolates were resistant to erythromycin and 31.58% (12/38) of isolates showed high MICs value of ≥ 64 μg/ml for tylosin, whereas only two isolates carried the macrolide resistance gene *mef(B)*, implying that novel determinants could play a part in such resistance. As for antimicrobials of fluoroquinolones and β-lactams, we found two isolates showed extremely high MIC values of >16 μg/ml for enrofloxacin, and 28.94% (11/38) of isolates are ampicillin resistance while eight isolates (21.05%) were positive for β-lactam resistance genes including *bla*_CTX–M–14_, *bla*_OXA–1_, and *bla*_ROB–1_ (Supplementary Table 2 and Supplementary Figure 3). The genetic environment of *bla*_ROB–1_ in *A. paragallinarum* showed similarity to the plasmid pB1000 found in *Glaesserella parasuis*. Besides, compared with the breakpoint of streptomycin in *A. paragallinarum*, seven isolates (18.42%) showed resistance to gentamicin with a MIC value of >16 μg/ml. The genetic environment of type II gene cluster composed of several aminoglycoside resistance genes (*ant*, *aac*, and *aph*) shared high sequence similarity for the multi-drug resistance plasmid pYMH5, which was homologous to broad host-range plasmid pLS88 from *H. ducreyi*. Notably, 13 of 38 *A. paragallinarum* isolates exhibited potentially resistant to colistin with MICs value of 32 μg/ml or greater, but colistin resistance genes were not found, indicating a novel mechanism contributed to this phenotype.

Strikingly, resistance genes were almost flanked by mobile genetic elements. Several resistance genes associated with MGEs combined into a large gene cluster were globally distributed in this study as shown in Figure 5. Additionally, we also depicted the VGs and their genetic environment in *A. paragallinarum*. All of *A. paragallinarum* were carried VGs *lpxC*, *manB/yhxB*, and *gmhA/lpcA*, which involved in lipooligosaccharide (LOS) and exopolysaccharide biosynthesis. Endotoxin biosynthesis genes *kdsA* were restricted to group B *A. paragallinarum*, suggesting that *kdsA* acted as a marker gene in comparison to group A *A. paragallinarum* (Supplementary Figure 3). Notably, 71.05% of phosphohexose isomerase genes (*gmhA/lpcA*) were flanked by integrases and insertion sequences (Figure 5B). The positive virulence and antibiotic resistance genes were list in Supplementary Table 3.

## Discussion

The resident microbiota plays a crucial role in eradicating the colonization of pathogens and rebuilding microbial defense systems to maintain homeostasis (Li et al., 2019). However, certain commensal bacteria have been found to be positively correlated with the growth of pathobionts during infections (Stacy et al., 2014). The symbiotic relationship between *A. paragallinarum* and commensal microbiota may compromise therapeutic efficacies and aggravate persistent infections (Rawson et al., 2020; Westblade et al., 2021). Besides, the species of commensal bacteria that contribute to such infection remains elusive. Here, we find that several species of commensal bacteria promote the growth of *A. paragallinarum*, including *Staphylococcus*, *Enterococcus*, and *Bacillus*. These commensals are mainly composed of Gram-positive bacteria, consisting with previous observations that Gram-positive commensals create suitable niches for Gram-negative pathogens under resource-limited conditions (Piccardi et al., 2019). Hence, commensals are important for the survival of *A. paragallinarum*, especially for these co-existed Gram-positive bacteria in the respiratory tract.

The hemolytic activity may play a crucial role in interspecies interactions. The lysis of RBCs by commensal subsequently releases intracellular contents particularly NAD^+^ (Mouahid et al., 1992) to favor the survival of opportunistic pathogens. The capability of hemolysis in *B. cereus*, *C. jeikeium*, *S. hyicus, S. aureus*, and *S. haemolyticus*, can disrupt host barriers to accelerate the replications of *A. paragallinarum* in respiratory tract. For example, *B. cereus* is capable to produce diverse enterotoxins like Nhe and Hbl (Cui et al., 2016; Zhu et al., 2016), to promote the survival and growth of *A. paragallinarum*. Similarly, toxins secreted by *Staphylococcus* spp. potentiate infections as well (Cohen et al., 2016). Therefore, commensal-mediated hemolysis probably contributes to the increased abundance of *A. paragallinarum.*

The antimicrobial resistant *A. paragallinarum* aggravate infections. *A. paragallinarum* show increasing resistance to antimicrobial agents that are recommended for the treatment of IC such as ampicillin and tetracycline (Nhung et al., 2017; Fauziah et al., 2021). We find that 28.94% (11/38) of *A. paragallinarum* isolates show resistance to ampicillin. Nevertheless, Heuvelink et al. (2018) test 44 field isolates originating from 25 outbreaks and find all isolates are sensitive to ampicillin. Besides, *tetB* is present in all *A. paragallinarum* isolates and locates in the Tn*10* transposon. The high level of tetracycline may promote the acquisition of mobile *tetB* gene in sensitive strains. Additionally, the genetic environment of Tn*10* is similar to the ICEs in *G. parasuis* (Figure 2), suggesting that the transferable interspecies gene *tetB* is undergoing within *Haemophilus* (Sun et al., 2020). Notably, several strains show high MIC values (≥32 μg/ml) of colistin, whereas no *mcr* genes are detected in *A. paragallinarum*, indicating that additional undetermined resistant elements may contribute to such phenotype. Conclusively, the resistance characteristics emerging in *A. paragallinarum* enable them to better survival *in vivo*.

## Conclusion

The composition and diversity of URT microbiota shift by the colonization of *A. paragallinarum*. Commensal bacteria particularly Gram-positive commensals show closely related to the presence of resistant *A. paragallinarum* and benefit the survival thereof. The commensal-mediated hemolysis further promotes the growth of *A. paragallinarum* and probably aggravates the infection. Therefore, targeting the population dynamics of commensal bacteria may be a promising therapeutic approach to treat IC.

## Data availability statement

The draft whole genome sequence assemblies in the study are deposited in the GenBank repository, accession number PRJNA836796 can found in the article/Supplementary material.

## Ethics statement

This animal study was reviewed and approved by the China Agricultural University. Written informed consent was obtained from the owners for the participation of their animals in this study.

## Author contributions

JJZ: conceptualization and writing—review and editing. YSC: methodology, validation, formal analysis, investigation, and writing—review and editing. YFW: methodology, validation, and formal analysis. KZ and YQW: conceptualization, methodology, and funding acquisition. All authors contributed to the article and approved the submitted version.
