# Supplementary material for: Commensal bacteria contribute to the growth of multidrug-resistant Avibacterium paragallinarum in chickens
Source: Front Microbiol. 2022 Nov 4;13:1010584. doi: 10.3389/fmicb.2022.1010584 (PMC9672371; doi:10.3389/fmicb.2022.1010584)
Supplement: Supplementary file 1 [file Data_Sheet_1.pdf]

Table S1 Detailed information about isolation, colocalization and satellitism profiles of commensal bacteria in URT of IC chickens.

Commensal bacteria	Isolation rate (%)	Satellitism[a]	Satellitism rate (%)	Colocalization rate (%)
***Acinetobacter*[b]**	**4.93**	**+**	**6.46**	**22.73**
A. lwoffii	2.78	+	3.65	13.64
A. schindleri	2.14	+	2.81	18.18
Bacillus	**16.27**	**+**	**21.35**	**40.91**
B. amyloliquefaciens	0.86	+	1.12	4.55
B. cereus	0.86	+	1.12	4.55
B. haynesii	3.85	+	5.06	22.73
B. licheniformis	0.21	+	0.28	0.00
B. pumilus	0.21	+	0.28	0.00
B. safensis	0.21	+	0.28	0.00
Bacillus sp.	1.07	+	1.40	9.09
B. subtilis	8.14	+	10.67	27.27
B. velezensis	0.64	+	0.84	0.00
B. wiedmannii	0.21	+	0.28	0.00
Corynebacterium	**4.28**	**+/-**	**1.12**	**31.82**
C. falsenii	2.14	-	0.00	27.27
C. geronticis	0.43	-	0.00	0.00
C. glutamicum	0.43	-	0.00	4.55
C. jeikeium	0.86	+	1.12	9.09
Corynebacterium sp.	0.21	-	0.00	0.00
C. vitaeruminis	0.21	-	0.00	0.00
Enterococcus	**16.49**	**+/-**	**17.13**	**77.27**
E. casseliflavus	0.43	-	0.00	9.09
E. cecorum	2.14	-	0.00	22.73
E. faecalis	13.06	+	17.13	63.64
E. faecium	0.21	-	0.00	0.00
E. gallinarum	0.64	-	0.00	4.55
Kocuria	**1.71**	**+/-**	**1.69**	**9.09**
K. kristinae	0.21	+	0.28	4.55
K. rosea	1.07	+	1.40	0.00
Kocuria sp.	0.43	-	0.00	4.55
Paenibacillus	**1.07**	**+/-**	**0.28**	**9.09**
P. barengoltzii	0.64	-	0.00	4.55
P. illinoisensis	0.21	+	0.28	0.00
P. polymyxa	0.21	-	0.00	4.55
Rothia	**5.57**	**+/-**	**6.18**	**27.27**
R. amarae	0.64	-	0.00	9.09
R. nasimurium	4.71	+	6.18	13.64

Rothia sp.	0.21	-	0.00	4.55
Staphylococcus	**33.19**	+	**43.54**	**72.73**
S. aureus	7.71	+	10.11	18.18
S. chromogenes	19.70	+	25.84	63.64
S. epidermidis	0.21	+	0.28	4.55
S. gallinarum	0.43	+	0.56	0.00
S. haemolyticus	0.64	+	0.84	4.55
S. hyicus	3.64	+	4.78	13.64
S. pseudintermedius	0.21	+	0.28	0.00
S. saprophyticus	0.21	+	0.28	0.00
S. sciuri	0.21	+	0.28	4.55
S. warneri	0.21	+	0.28	0.00
**Others genera[c]**	**16.49**	**+/-**	**2.24**	**/**
Aerococcus viridans	0.21	-	0.00	4.55
Avibacterium paragallinarum	8.14	/	/	/
Carnobacterium sp.	0.21	-	0.00	4.55
Enterobacter sp.	0.21	-	0.00	0.00
Escherichia coli	0.64	-	0.00	4.55
Gallibacterium anatis	3.43	-	0.00	18.18
Lysinibacillus sp.	0.21	+	0.28	0.00
Pseudomonas aeruginosa	1.71	-	0.00	9.09
Stenotrophomonas maltophilia	0.43	+	0.56	0.00
Streptococcus pluranimalium	1.07	+	1.40	13.64
Vibrio sp.	0.21	-	0.00	0.00

Note: (a) /, not application; +, positive for satellitism, -, negative for satellitism, +/-, part of species were positive for satellitism and the rest of these were opposite. (b) The bacterial genera were in bold and filled with grey. (c) The "Others genera" group contained the bacterial genera only had a single specie and listed the full species name in this table.

Table S2 The profile of bacterial composition of healthy chickens.

Commensal bacteria	Number of isolates	Isolation rate (%)
Escherichia	31	35.6%
Escherichia coli	31	35.6%
Klebsiella	18	20.7%
Klebsiella pneumoniae	15	17.2%
Klebsiella quasivariicola	1	1.1%
Klebsiella variicola	2	2.3%
Rothia	16	18.4%
Rothia amarae	5	5.7%
Rothia nasimurium	11	12.6%
Enterococcus	13	14.9%
Enterococcus casseliflavus	1	1.1%
Enterococcus faecalis	5	5.7%
Enterococcus faecium	4	4.6%
Enterococcus gallinarum	2	2.3%
Enterococcus hirae	1	1.1%
Shigella	3	3.4%
Shigella sonnei	3	3.4%
Bacillus	2	2.3%
Bacillus licheniformis	2	2.3%
Kocuria	2	2.3%
Kocuria rosea	2	2.3%
Staphylococcus	1	1.1%
Staphylococcus saprophyticus	1	1.1%
Streptococcus	1	1.1%
Streptococcus lutetiensis	1	1.1%

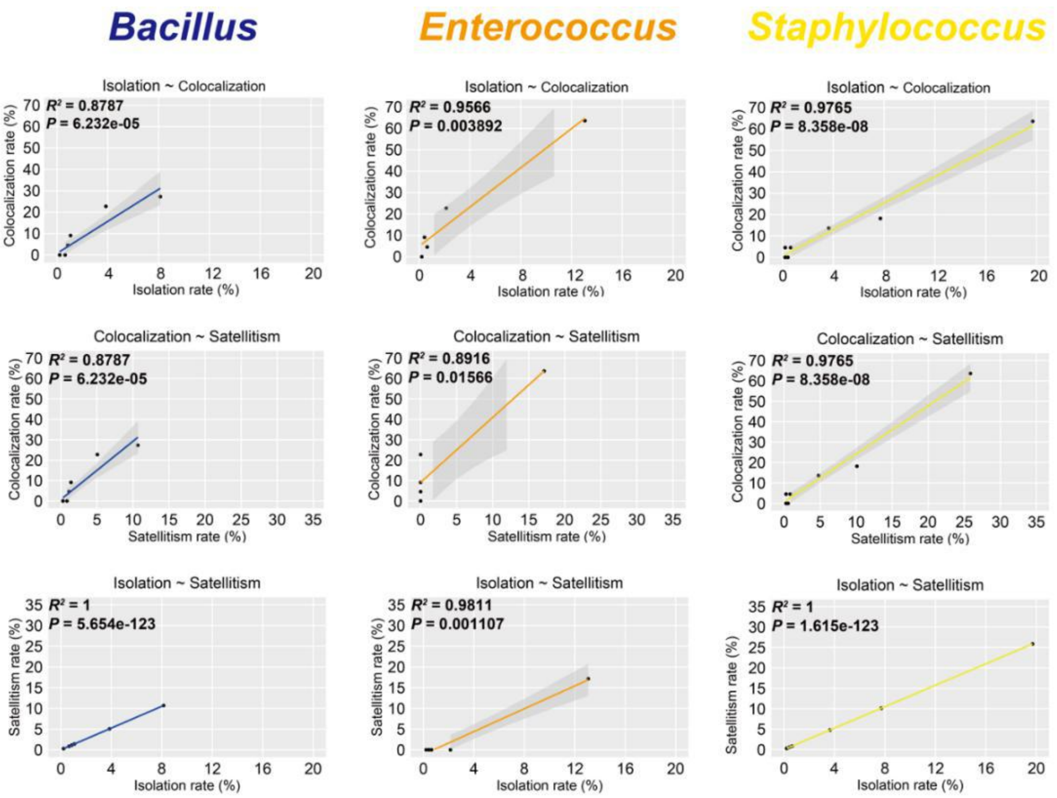

Figure S1 Isolation correlated with colocalization and satellitism in *Bacillus*, *Enterococcus* and *staphylococcus*. The correlation between isolation and satellitism in *Bacillus* and *staphylococcus* are perfectly fit to liner model. The grey shaded areas represented the 95% confidence intervals.

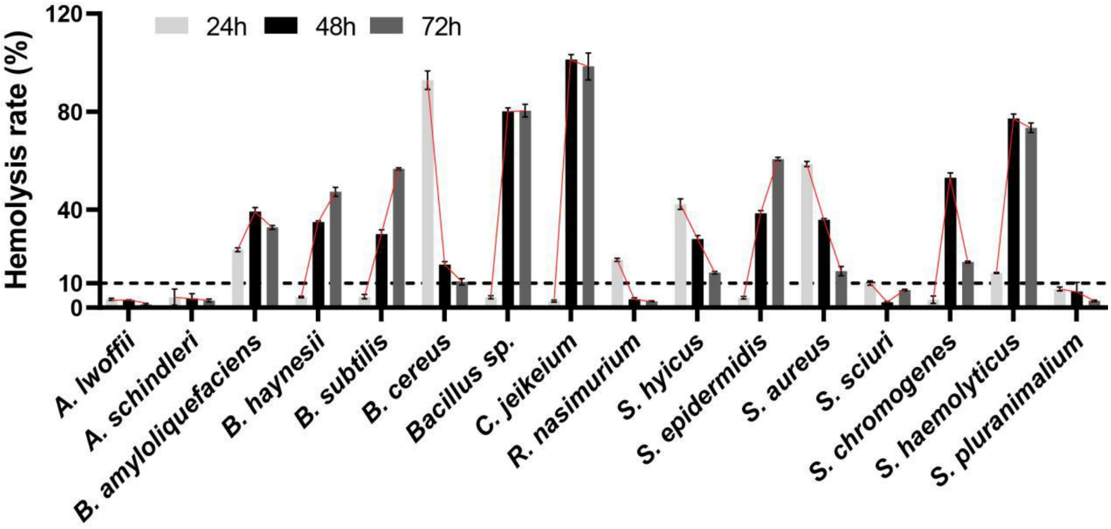

Figure S2 Hemolysis changes of selected commensals in broth cultivation. Hemolytic activity in 16 commensals were depicted at 24, 48 and 72h, respectively. The red lines represented the dynamic changes of hemolysis.

Table S2 MIC values of *A. paragallinarum*

Isolates	Chloramphenicols	Lincosamides	Macrolides		Tetracyclines	β -lactam					Fluoroquinolone	Aminoglycoside	Lipopeptide
	Florfenicol (FFC)	Clindamycin (CLI)	Erythromycin (ERY)	Tylosin (TIL)	Tetracycline (TET)	Penicillin (PEN)	Ampicillin (AMP)	Cefuroxime (CFX)	Oxacillin (OXA)	Meropenem (MEM)	Enrofloxacin (ENR)	Gentamicin (GEN)	Colistin (COL)
AV1	8	>16	16	64	32	4	32	0.25	>16	<0.03	1	4	32
AV2	16	>16	16	64	32	4	64	0.5	>16	<0.03	1	16	32
AV3	8	>16	16	64	32	2	64	0.5	>16	<0.03	1	4	64
AV4	8	>16	16	64	32	4	64	1	>16	0.25	0.5	16	8
AV5	8	>16	16	64	32	4	32	0.25	>16	<0.03	1	4	32
AV6	8	>16	16	64	32	4	32	1	>16	<0.03	1	8	32
AV7	8	>16	8	64	32	4	16	0.5	>16	<0.03	1	4	32
AV8	2	>16	8	4	32	8	0.5	0.25	>16	<0.03	1	4	32
AV9	2	>16	8	4	32	4	0.5	0.25	>16	<0.03	1	4	32
AV10	2	>16	8	4	32	8	0.5	1	>16	<0.03	1	4	32
AV11	4	2	1	1	2	2	8	32	>16	0.03	8	>16	0.125
AV12	4	2	4	2	2	2	8	32	>16	0.03	8	>16	0.125
AV13	4	16	8	16	32	2	2	0.12	>16	0.03	1	4	32
AV14	2	16	8	16	16	1	0.5	0.12	>16	0.03	2	>16	32
AV15	4	8	4	8	8	2	0.5	0.25	>16	0.03	1	4	16
AV16	2	>16	16	4	32	4	0.5	2	>16	<0.03	1	>16	32
AV17	4	16	8	16	16	1	1	0.12	>16	0.03	1	4	16
AV18	4	16	8	8	16	1	0.25	32	16	0.03	1	16	>64
AV19	<0.5	>16	16	>64	1	2	0.5	>128	>16	<0.03	0.5	1	2
AV20	<0.5	>16	16	>64	8	>64	16	0.25	>16	<0.03	8	<0.12	0.12

AV21	<0.5	>16	16	0.5	8	0.25	<0.12	0.25	>16	<0.03	<0.03	<0.12	2
AV22	<0.5	>16	16	0.5	0.5	2	<0.12	0.25	>16	<0.03	0.5	<0.12	2
AV23	<0.5	16	8	4	32	2	<0.12	0.25	4	<0.03	2	0.25	2
AV24	<0.5	16	8	2	64	16	1	0.25	>16	<0.03	1	0.25	4
AV25	<0.5	16	16	32	64	16	1	0.5	>16	<0.03	1	<0.12	4
AV26	<0.5	>16	16	32	32	16	1	0.25	>16	<0.03	1	<0.12	4
AV27	<0.5	>16	4	16	64	2	0.5	0.5	>16	<0.03	1	0.5	4
AV28	<0.5	8	8	4	64	4	0.5	0.5	>16	<0.03	2	0.5	16
AV29	>256	16	16	64	32	4	0.25	0.5	>16	<0.03	2	0.25	4
AV30	>256	>16	16	64	1	2	<0.12	<0.12	>16	<0.03	0.5	<0.12	4
AV31	<0.5	>16	16	16	16	2	<0.12	<0.12	>16	<0.03	0.5	<0.12	2
AV32	<0.5	>16	8	16	1	2	0.25	0.25	>16	<0.03	0.5	<0.12	2
AV33	<0.5	>16	8	16	32	2	0.5	0.25	>16	<0.03	0.5	<0.12	2
AV34	<0.5	16	8	8	1	2	0.5	<0.12	>16	<0.03	0.5	<0.12	2
AV35	<0.5	16	4	8	32	4	64	0.25	16	<0.03	0.5	<0.12	2
AV36	<0.5	>16	8	64	32	>64	64	0.25	16	<0.03	>16	<0.12	0.12
AV37	<0.5	16	16	4	32	>64	64	0.25	>16	<0.03	>16	0.25	0.12
AV38	<0.5	>16	2	32	32	2	0.5	0.25	>16	<0.03	0.5	0.25	2
Resistance rate (%)[a]	ND	ND	84.21	ND	73.68	50	28.94	ND	ND	0	ND	ND	ND
MIC_{50}[b]	2	>16	8	16	32	2	0.5	0.25	>16	<0.03	1	0.5	4
MIC_{90}[b]	8	>16	16	64	64	16	64	2	>16	0.03	8	16	32

Note: (a) ND, not defined MIC breakpoint. Resistance rate represents the proportion of antimicrobial resistance isolates; (b) MIC_{50} and MIC_{90} are defined as the concentration of the antibiotics able to inhibit the growth of 50% and 90% of the isolates in the test population, respectively.

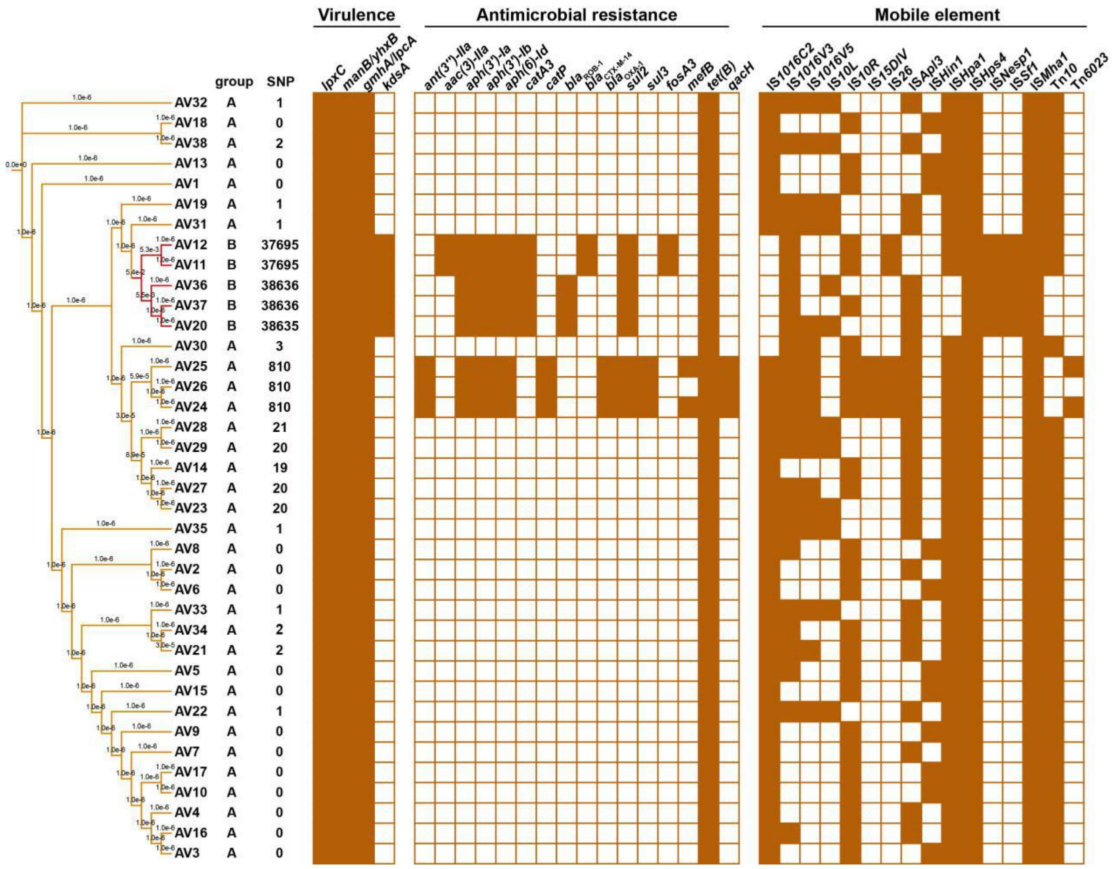

Figure S3 Virulence, antimicrobial resistance and mobile element profiles of *A. paragallinarum.* The classification of genomic group was determined by genomic SNP analysis. Red lines in phylogenetic tree indicates distinct genomic relationship.

Table S3 Positive virulence and antimicrobial resistance genes in this study.

**Gene**	**Accession**[a]	**Product**
aac(3)-IIa	X51534	Aminoglycoside-(3)-N-acetyltransferase
ant(3")-Ia	X02340	Aminoglycoside 3" adenyltransferase
aph(3')-Ia	V00359	Aminoglycoside phosphotransferase
aph(3")-Ib	AF024602	Streptomycin resistance protein
aph(6)-Id	M28829	Streptomycin resistance protein
$bla_{\text{CTX-M-14}}$	AF252622	Beta-lactamase CTX-M-14
$bla_{\text{OXA-1}}$	HQ170510	Beta-lactamase OXA-1
$bla_{\text{ROB-1}}$	DQ840517	Beta-lactamase ROB-1
catA3	X07848	Type III chloramphenicol acetyltransferase
catP	U15027	Chloramphenicol acetyltransferase
fosA	AB522970	Fosfomycin resistance protein
mef(B)	FJ196385	Macrolide efflux pump
sul2	AY034138	Dihydrofolate reductase
qacH	AAX56371	Multidrug efflux pump
sul3	AJ459418	Dihydropteroate synthase
tetB	AF326777	Tetracycline efflux protein
lpxC	WP_005630366	UDP-3-O-acyl-N-acetylglucosamine deacetylase
manB/*yhxB*	WP_012340250	Phosphomannomutase
gmhA/*lpcA*	WP_005630454	Phosphoheptose isomerase

Note: (a) The GenBank accession number of each reference gene.
